# Oxidative stress as a risk factor for osteoporosis in elderly Mexicans as characterized by antioxidant enzymes

**DOI:** 10.1186/1471-2474-8-124

**Published:** 2007-12-19

**Authors:** Martha A Sánchez-Rodríguez, Mirna Ruiz-Ramos, Elsa Correa-Muñoz, Víctor Manuel Mendoza-Núñez

**Affiliations:** 1 Unidad de Investigación en Gerontología, Facultad de Estudios Superiores Zaragoza, Universidad Nacional Autónoma de México (UNAM), México D.F., México; 2 Batalla 5 de mayo s/n, esq. Fuerte de Loreto, Col. Ejército de Oriente, 09230 México, DF., México

## Abstract

**Background:**

Oxidative stress (OxS) has recently been linked with osteoporosis; however, we do not know the influence of OxS as an independent risk factor for this disease.

**Methods:**

We conducted a case-control study in 94 subjects ≥60 years of age, 50 healthy and 44 with osteoporosis. We measured total antioxidant status, plasma lipid peroxides, antioxidant activity of superoxide dismutase and glutathione peroxidase (GPx), and calculated the SOD/GPx ratio. Bone mineral density was obtained at the peripheral DXA in calcaneus using a portable Norland Apollo Densitometer^®^. Osteoporosis was considered when subjects had a BMD of 2.5 standard deviations or more below the mean value for young adults.

**Results:**

GPx antioxidant activity was significantly lower in the group of subjects with osteoporosis in comparison with the group of healthy subjects (*p *< 0.01); in addition, the SOD/GPx ratio was significantly higher in the group of individuals with osteoporosis (*p *< 0.05). In logistic regression analysis, we found OxS to be an independent risk factor for osteoporosis (odds ratio [OR] = 2.79; 95% confidence interval [95% CI] = 1.08–7.23; *p *= 0.034).

**Conclusion:**

Our findings suggest that OxS is an independent risk factor for osteoporosis linked to increase of SOD/GPx ratio.

## Background

Oxidative stress (OxS) is a biochemical disequilibrium propitiated by excessive production of free radicals (FR) and reactive oxygen species (ROS), which provoke oxidative damage to biomolecules and which cannot be counteracted by antioxidative systems. This biochemical alteration has been linked with aging and more of 100 chronic-degenerative diseases, among which osteoporosis is found [1,2].

Thus, epidemiologic studies on osteoporosis should consider OxS, in addition to risk factors linked with lifestyles, hormonal changes, and aging [3-5]. It has been demonstrated recently that FR intervene in bone resorption, promoting osteoclastic differentiation in such a manner that bone resorption is increased with OxS [6-8].

Similarly, experimental studies have shown a diminution in antioxidant activity in patients with osteoporosis [9,10]. In this regard, Arjmandi et al. (2002) demonstrated that administration of vitamin E has a beneficial effect on bone quality in old rats [11]; nevertheless, the association between OxS and bone mineral density (BMD) in humans has scarcely been approached.

In an exploratory study, our investigation group found a negative correlation between BMD and total antioxidant status (TAS) linked with serum levels low in glutathione peroxidase (GPx) [12,13]; notwithstanding this, the influence of OxS as an independent risk factor is unknown, considering the contribution of the additional risk factors linked with lifestyles, age, and sex. Therefore, the objective of this study was to determine the relationship of OxS as an independent risk factor for osteoporosis in a population of elderly adults.

## Methods

### Study subjects

We carried out a case-control study in a convenience sample of 94 subjects ≥ 60 years of age, 50 healthy, 20 men and 30 women (mean age 67.9 ± 6.5 years) and 44 with osteoporosis, 19 men and 25 women (mean age 69.7 ± 7.3 years). The subjects were community-dwelling Mestizo Mexican elderly living in Mexico City for 10 years or more. Informative brochures were distributed in the community specifying the objectives of the study and admission criteria.

All the women had intact uterus and the mean age in which their menopause began is the same between the two study groups. None of the subjects studied had been taking antioxidant supplementation (vitamins or minerals), hormone replacement therapy or antiosteoporotic medication for at least 6 months prior to the study, none had acute or chronic diseases, or was receiving prescription medications.

Both groups were well-nourished, Mini Nutritional Assessment (MNA) score was > 23.5, and caloric intake was between 2,000 and 2500 kcal per day, and the alimentation had the nutrients requirements (protein, fat, carbohydrate, vitamins and minerals) between Recommended Dietary Allowance (RDA) measured by 24-h dietary recalls and serum albumin > 35 g/L [14,15].

The subjects agreed to participate in the study after giving their informed consent. The Ethics Committee of the Universidad Nacional Autónoma de México (UNAM) Zaragoza Campus approved the research protocol for this study.

### Measurements

The following anthropometric measurements were obtained: weight, height, body mass index (BMI). It was considered as overweight when BMI ≥ 27 [16].

Weight was measured while the subject was wearing underwear and a clinical smock and in a fasted state (after evacuation). A Torino^® ^scale (Tecno Lógica, Mexicana, México, TLM^®^) was used, calibrated before each weight measurement. Height was obtained with an aluminum cursor stadiometer graduated in millimeters. The subject was barefoot, back, and head in contact with the stadiometer in Frankfurt horizontal plane. BMI was calculated by dividing weight (in kilograms) by height (in square meters).

Bone mineral density (BMD) was obtained at the peripheral DXA in calcaneus using a portable Norland Apollo Densitometer^®^. Osteoporosis was considered when subjects had a BMD of 2.5 standard deviations (SD) or more below the mean value for young adults (T score, ≥2.5) [17].

Blood samples were collected after a 12-h fasting period by venopuncture and placed in vacutainer/siliconized test tubes containing a separating gel and no additives, and heparin as anticoagulant agent (Becton-Dickinson, Mexico City, Mexico). Blood samples containing heparin were analyzed using a hemoglobin test protocol (including hemoglobin, hematocrit, and leukocyte counts). The following serum quantifications were conducted: glucose; urea; creatinine; urate; albumin; cholesterol; triglycerides, and cholesterol high-density lipoproteins (HDL). These tests were used as screening measurements for diagnosis of clinically healthy subjects.

Hemoglobin levels were measured by cyanomethahemoglobin reaction procedure (cut-off points: in males, 12.17–17.26 g/dL, and in females, 11.48–16.25 g/dL). Hematocrit levels were assessed by microhematocrit procedure (cut-off points: males, 38–52%, females, 36–51%). Leukocyte count was done using Newbauer Chamber procedure (cut-off points: 3,500–10, 650/mm^3^).

All spectrophotometric tests were determined using an UV-visible spectrophotometer (Shimadzu, Columbia, MD, USA). Specifically, glucose levels were measured with glucose oxidase method (cut-off points: 63–120 mg/dL), urea levels were measured with Berthelot urease method (cut-off points: 9.5–47.0 mg/dL), creatinine levels by Jaffe method without deproteinization (cut-off points: males, 0.3–1.5 mg/dL, females, 0.3–1.3 mg/dL), and urate levels by uricase colorimetric method (cut-off points: males, 2.9–8.88 mg/dL, females, 2.5–8.7 mg/dL). Albumin levels were measured by bromocresol green technique (3.23–4.03 g/dL).

Cholesterol was analyzed using CHOD-PAP technique (cut-off points: 168–200 mg/dL) and triglycerides by GPO-Trinder technique (cut-off points: 89–190 mg/dL), whereas HDL were assessed employing the same technique for cholesterol after precipitation of low and very-low lipoproteins using a phosphotungstic acid/magnesium chloride solution (cut-off points: 42–77 mg/dL).

All reagents employed in biochemical tests were obtained from Randox Laboratories, Ltd. (Crumlin, Co, Antrim, UK); cut-off points for reference values for Mexican elderly persons were determined at the Gerontologic Clinical Research Laboratory of the Universidad Nacional Autónoma de México (UNAM) Zaragoza Campus in Mexico City [18].

Blood samples containing heparin were subjected to plasma total antioxidant status (TAS), activity of red blood cell (RBC) superoxide dismutase (SOD) and glutathione peroxidase (GPx), and plasma TBARS assay. Artefactual formation of TBARS in the samples was prevented by adding 10 μL of 2-mM butylated hydroxytoluene (BHT) in ethanol at 95% immediately after centrifugation.

Total antioxidant status was carried out using ABTS+ (2,2'-azidodiethylbenzothiazolin sulphanate) radical formation kinetics (Randox Laboratories, Ltd). The presence of antioxidants in plasma suppressed the bluish-green staining of the ABTS^+ ^cation, which was proportional to the antioxidant concentration level. Kinetics is measured at 600 nm.

The method employs xanthine and xanthine oxidase (XOD) to generate superoxide radicals, which react with 2-(4-iodophenyl)-3-(4-nitrophenol)-5-phenyltetrazolium chloride (INT) to form a red formazan dye. SOD activity was measured by degree of inhibition of the reaction (Randox Laboratories, Ltd). Kinetics was measured at 505 nm.

GPx catalyses oxidation of glutathione (GSH) by cumene hydroperoxide, in the presence of glutathione reductase (GR) and NADPH; oxidized glutathione (GSSG) is immediately converted into the reduced form with concomitant oxidation of NADPH to NADP^+^. Decrease in absorbance at 340 nm is measured (Randox Laboratories, Ltd).

We used thiobarbituric acid reacting substances (TBARS) assay. It was performed as described by Jentzsch et al. (1996) [19]. In the TBARS assay, one molecule of malondialdehyde (MDA) reacts with two molecules of thiobarbituric acid (TBA) with production of a pink pigment with absorption at 535 nm. Amplification of peroxidation during the assay is prevented by the addition of the chain-breaking antioxidant BHT. Plasma (400 μL) or MDA standard (0.2–4 μmol/L) prepared by hydrolysis of 1,1,3,3-tetramethoxypropane (TMP) (Sigma Chemical Co, St. Louis, MO, USA) was mixed with 400 μL orthophosphoric acid (0.2 mol/L) (Sigma Chemical Co.) and 50 μL BHT (2 mmol/L) (Sigma Chemical Co.) in 12 × 75 mm tubes. Then we added 50 μL TBA reagent (0.11 mol/L in 0.1 mol/L NaOH) (Fluka Chem., Buchs, Switzerland) and mixed the contents; subsequently, the contents were incubated at 90°C for 45 min in a water bath. The tubes were put on ice to stop the reaction. TBARS were extracted once with 1000 μL n-butanol (Sigma Chemical Co.). The upper butanol phase was read at 535 nm and 572 nm to correct for baseline absorption in UV-visible spectrophotometer (Shimadzu, Columbia, MD, USA). MDA equivalents (TBARS) were calculated using the difference in absorption at two wavelengths and quantification was done with calibration curve.

The intra- and inter-assay variation coefficients were less 5% in all the determinations.

The within-run precision for the markers were as follows: LPO by TBARS assay 4.6%; erythrocyte SOD 3.8%; GPx and TAS with Randox Laboratories 6% and 4.3%, respectively. We calculated SOD/GPx ratio.

Alternative cut-off values for each parameter were defined on the basis of the 90^th ^percentile of young healthy subjects. A stress score [SS] ranging from 1 to 6 was used, representing the severity of biomarkers modifications; a score 1 was given to each value higher or lower than the cut-off. We categorize subjects as follows according to their scale in SS: with OxS if SS was > 3, and without OxS if SS was 0–3 [20,21].

Risk factors were measured: sex (female), age (≥ 70 years), oxidative stress (SS > 3), cigarette smoking (≥ 1 year in the present), alcohol intake (≥ 2 cups/day) and overweight (BMI ≥ 27).

### Statistical analysis

Data were processed by use of standard statistical software SPSS 10.0 (SPSS Inc. Chicago, IL, USA). Descriptive statistics are means ± standard deviations (SD). Results were analyzed using the Student's *t*-test and linear correlation analyses. Also were performed odds ratio (OR) of logistic regression analysis with 95% confidence interval (CI) and pseudo R-square (R^2^) [22]. A *p*-value < 0.05 was considered significant.

## Results

Average age, body mass index (BMI), and biochemical and hematologic parameters did not show statistically significant differences between the group of healthy subjects and that of those with osteoporosis (Table 1). Concerning OxS biological markers, we found that LPO did not show differences statistically significant (healthy, 0.258 ± 0.09 vs. osteoporosis, 0.296 ± 0.14 μmol/L; *p *> 0.05). However, GPx antioxidant activity was significantly lower in the group with osteoporosis in comparison with the healthy group of subjects (healthy, 7,039 ± 2,724 vs. osteoporosis, 5,399 ± 2,359 U/L; *p *< 0.01). Additionally, the SOD/GPx ratio was significantly higher in the group with osteoporosis (healthy, 0.028 ± 0.01 vs. osteoporosis, 0.036 ± 0.01; *p *< 0.05), the remainder of the parameters not showing significant changes (Table 2).

**Table 1:** Age, body mass index, and biochemical characteristics

	**Healthy **(n = 50)	**Osteoporosis **(n = 44)
Age (years)	67.9 ± 6.5	69.7 ± 7.3
Body Mass Index (BMI) (kg/m^2^)	29.30 ± 3.9	27.85 ± 4.7
Glucose (mg/dL)	103 ± 13.8	94 ± 13.2
Urea (mg/dL)	29 ± 3.9	28 ± 4.7
Creatinine (mg/dL)	0.82 ± 0.26	0.89 ± 0.48
Urate (mg/dL)	5.0 ± 1.6	4.6 ± 1.4
Cholesterol (mg/dL)	222 ± 43	218 ± 53
Triglycerides (mg/dL)	203 ± 102	180 ± 72
HDL (mg/dL)	47 ± 10.5	50 ± 14.7
Albumin (g/dL)	4.3 ± 0.5	4.2 ± 0.5
Hemoglobin (g/dL)		
Female	14.8 ± 1.0	14.1 ± 1.1
Male	15.7 ± 1.5	15.8 ± 1.7
Hematocrit (%)		
Female	46 ± 2.9	44 ± 3.6
Male	49 ± 4.2	48 ± 5.0
Total leukocytes/mm^3^	6359 ± 1456	6459 ± 2273

Mean value ± SD (standard deviation), t-test p > 0.05.

**Table 2:** Oxidative stress markers in healthy and osteoporosis subjects

	**Healthy **(n = 50)	**Osteoporosis **(n = 44)
Lipoperoxides (LPO) (μmol/L)	0.258 ± 0.09	0.296 ± 0.14
Superoxide dismutase (SOD) (UI/L)	167 ± 10.7	165 ± 7.4
Glutathione peroxidase (GPx) (UI/L)	7039 ± 2724	5399 ± 2359*
Total antioxidant status (TAS) (mmol/L)	1.03 ± 0.21	0.96 ± 0.18
SOD/GPx ratio	0.028 ± 0.01	0.036 ± 0.01^†^

Mean values ± SD; t-test, *p < 0.01, ^†^p < 0.05.

Similarly, we observed a positive correlation between BMD with GPx antioxidant activity (r = 0.31; *p *< 0.01), TAS (r = 0.20; p < 0.05) and BMI (r = 0.19; p < 0.05), and a negative correlation with the SOD/GPx ratio (r = -0.30; *p *< 0.01). At the same time, there is a negative correlation between GPx and LPO (r = -0.22; p < 0.05) (Table 3).

**Table 3:** Correlation between age, BMI, oxidative stress markers and BMD

		T SCORE	LPO	SOD	GPX	TAS	Age	BMI	SOD/GPx
r value	T SCORE	1.000	-0.090	-0.009	0.316	0.198	-0.111	0.191	-0.309
	LPO		1.000	-0.106	-0.219	-0.060	-0.068	0.138	0.099
	SOD			1.000	0.006	0.136	-0.021	-0.081	0.061
	GPX				1.000	0.205	0.012	0.126	-0.885
	TAS					1.000	0.078	0.059	-0.161
	Age						1.000	0.077	-0.044
	BMI							1.000	-0.241
	SOD/GPx								1.000
									
Sig. (1-tailed)	TSCORE		0.215	0.467	0.002	0.039	0.163	0.045	0.003
	LPO			0.175	0.025	0.300	0.276	0.111	0.191
	SOD				0.478	0.115	0.425	0.236	0.295
	GPX					0.034	0.457	0.133	< 0.0001
	TAS						0.245	0.300	0.077
	Age							0.250	0.351
	BMI								0.016

Linear correlation analyses.

Regard risk factors, we found a higher percentage of osteoporosis in women, smokers and elderly with OxS, and lower in overweight individuals (Table 4).

**Table 4:** Frequency of risk factors to osteoporosis by study group

**Variable**	**Osteoporosis **(n = 44)	**Normal **(n = 50)
Gender		
Female	36 (82%)	34 (68%)
Male	8 (18%)	16 (32%)
Age		
60 – 69 years	21 (48%)	31 (62%)
≥ 70 years	23 (52%)	19 (38%)
Smoke		
Positive	6 (14%)	3 (6%)
Negative	38(86%)	47(94%)
Alcohol ingestion		
Positive(≥ 2 cups/day)	17 (38%)	13 (26%)
Negative	27 (62%)	37 (74%)
Weight		
Overweight (BMI ≥ 27)	23 (52%)	34 (68%)
Normal weight	21 (48%)	16 (32%)
Oxidative stress		
Positive	30 (68%)*	19 (38%)
Negative	14 (32%)	31(62%)

*χ^2 ^test, p < 0.01.

In agreement with the previously mentioned observations, we found an OxS frequency of 68% in the group of subjects with osteoporosis in contrast with 38% in healthy-subject group. In logistic regression analysis, we found that OxS is an independent risk factor to osteoporosis (OR = 2.79; 95% CI = 1.08–7.23; *p *= 0.034), while we similarly observed that sex (female) and age (≥70 years) are risk factors with OR of 4.47 and 3.45 respectively, besides the overweight (BMI ≥27) is a protective factor (OR = 0.36; 95% CI = 0.14–0.93; *p *< 0.05) (Table 5).

**Table 5:** Risk factors to osteoporosis

	**OR**	**CI**	**P value **
Sex (female)	4.47	1.25 – 16.02	0.022
Age (≥ 70 years)	3.45	1.21 – 9.81	0.020
Oxidative stress	2.79	1.08 – 7.23	0.034
Cigarette smoking	3.18	0.62 – 16.29	0.165
Alcohol intake (≥ 2 cups/day)	2.16	0.77 – 6.00	0.140
Overweight (BMI ≥ 27)	0.36	0.14 – 0.93	0.035

Logistic regression, R^2 ^= 0.255, p < 0.01; OR = odds ratio; CI = confidence interval

## Discussion

Oxidative stress measurement in humans with possibilities of clinical application is at present in a process of transition. Over the past several years, great advances have been made in the simplification of reliable diagnostic tests for evaluating certain OxS biological markers, such as the measurement of LPO, GPx, SOD, catalase (CAT), total antioxidant status (TAS), DNA damage, antioxidant vitamins (A, C, and E), and minerals (zinc, selenium), among others [23-25]. Nonetheless, integral clinical interpretation has been not been considered concerning the parameters' abnormal values, taking into consideration that OxS is a dynamic and complex process.

In this respect, our investigative group has developed a construct for a more integral measurement of OxS, taking the different components into consideration [19,20]. According to this construct, in our study we found OxS frequency to be nearly double in the group of subjects with osteoporosis compared with that of the healthy-subject group (osteoporosis, 68% vs. healthy, 38%; *p *< 0.05), this congruent with scientific evidence associating OxS with the etiology and physiopathology of osteoporosis [2,6,7,9].

Regarding OxS biochemical markers, it has been demonstrated that subjects with osteoporosis exhibit significantly higher LPO [2,6,26]. However, it has also been reported that there are no significant differences in the serum levels of subjects with and without osteoporosis in terms of this biological marker [9]. In our study, we did not find significatives differences between LPO levels in the group of subjects with osteoporosis compared with the healthy-subject group. Inconsistencies in the relationship between LPO and BMD could be due to the biological marker applied. In these circumstances, we must consider that F2-isoprostanes is a better marker than TBARS, which was applied in our study [2].

Oxidative stress is a dynamic process, and to those subjects having high LPO does not necessarily mean that these have OxS and vice versa. This because high LPO can be compensated for by the antioxidant activity of SOD, GPx, and CAT enzymes, as well as by antioxidant vitamins, among other elements involved in OxS; similarly, if low LPO coexist with very low antioxidant activity, the subject could have OxS [27-29]. Although

On the other hand, in this work we found that GPx antioxidant activity was significantly lower in the group with osteoporosis in comparison with the healthy group, in addition to a positive correlation between GPx and BMD (*r *= 0.31; *p *< 0.001), which is consistent with that reported by other authors [6,9,26]. Nevertheless, we found no statistically significant differences in SOD antioxidant activity between the groups with and without osteoporosis, this contrary to that reported by other authors [6,9], probably due to the previously mentioned fact that OxS is a dynamic and complex process.

A relevant finding in our study comprised the significantly higher value of the SOD/GPx ratio in the group of subjects with osteoporosis. In this respect, the imbalance propitiated by greater SOD activity with respect to GPx favors the increase in H_2_O_2 _levels, and consequently greater OxS [30,31]. Concerning this, it has been demonstrated that high H_2_O_2 _levels favor the differentiation of osteoblastic cells to osteoclasts and inhibit the differentiation of osteoblastic cells to osteoblasts, thus propitiating an accentuated diminution of BMD [6-8]. Although, we did not measure in this study the bone resorption or bone formation, our findings can be explained though this mechanism.

With respect to risk factors for osteoporosis, some authors have reported that age (≥70 years), sex (female), low weight (BMI ≤17), abundant intake of alcoholic beverages (≥2 drinks per day), smoking, drug intake (glucocorticoid therapy, aromatase inhibitors, androgen deprivation therapy), and a sedentary lifestyle, among others, are associated with an increase in the incidence of this disease [32-35]. In this regard, in our study we found that sex (female) and age (≥70 years) are significant risk factors for osteoporosis, in agreement with what has been reported by other authors [34,36]. In this regard, it has been pointed out that premenopausal hormonal decline increases osteoporosis incidence in women, and that aging propitiates bone fragility [37].

On the other hand, overweight (body mass index [BMI] ≥ 27) has been reported as a protective factor for osteoporosis [38], which was observed in our study; in this respect, an increase in the amount of biologically available estrogens has been observed, this due to the conversion of androstenedione into estrone in adipose cells, and a decrease in the concentration of sex hormone-binding globulins [39]. Likewise, a positive relationship has also been found between leptin and bone-specific alkaline phosphatase in elderly women and men, suggesting that BMI-linked leptin levels exert an influence on osteoblast activity in both sexes [40], although no relationship has been reported between leptin levels and bone mass [41,42]; thus, the role of leptin on BMD is not clear.

There must be considered as limitations of the study that BMD was not measured with gold standard" (DXA measurements of the spine or proximal femur), it was used instead peripheral-calcaneus DXA; nevertheless, several studies have shown that this method has a 90% sensitivity and 90% specificity for osteoporosis at the hip or spine [17,43]. Besides, this study is a cross-sectional and the sample is not representative; therefore it is necessary to carry out prospective studies with representatives samples to confirm our findings.

## Conclusion

The most relevant finding of the present study comprised having found evidence through multivariate analysis that OxS is an independent risk factor for osteoporosis, supporting the hypothesis that links OxS with the etiology and physiopathology of this disease. Therefore, the development of controlled clinical assays could be justified for evaluating the usefulness of diets and/or antioxidant supplements in complementary or preventive management of osteoporosis.

## Competing interests

The author(s) declare that they have no competing interests.

## Authors' contributions

MASR participated in the design of the study and biochemical analysis, MRR participated in the design of the study and biochemical analysis. ECM recruited participants, participated in the subject interviews, coded transcripts, and anthropometric measures. VMM conceived and designed the study, drafted the manuscript. All authors read and approved the final manuscript.

## Pre-publication history

The pre-publication history for this paper can be accessed here:


